# Surgical repair of post myocardial infarction ventricular septal defect: a retrospective analysis of a single institution experience

**DOI:** 10.1186/s13019-023-02418-8

**Published:** 2023-11-10

**Authors:** Jian Shi, Jeremy Y. Levett, Haiyu LV, Guoan Zhang, Sha Wang, Tao Wei, Zhikun Wang, Xi Zhang, Dawei Feng, Kan Wang, Qiang Liu, Dominique Shum-Tim

**Affiliations:** 1 Department of Cardiac Surgery, Shaanxi Provincial People’s Hospital, Xi’an, China; 2 Division of Cardiac Surgery, Department of Surgery, McGill University Health Centre, McGill University, Montreal, QC Canada

**Keywords:** Acute Myocardial Infarction, Ventricular septal defect, Surgical Repair, Mortality

## Abstract

**Introduction:**

Ventricular septal defect (VSD) is a mechanical complication of acute myocardial infarction (MI) with a very high mortality, despite advances in surgical and circulatory support. The tremendous hemodynamic disturbance and the severely fragile myocardium render surgical repair a great challenge. The optimal time of surgical repair with or without circulatory support is still controversial.

**Objective:**

The aim of this study is to review our experience with early surgical repair of post-MI VSD in a single major cardiac institution in China.

**Methods:**

From January 2013 to October 2020, 9consecutive patients presented to our emergency department with a diagnosis of post-MI VSD. Among them, 8 were male, and the mean age was 58 ± 7years. The mean VSD size was 22.5 ± 5.7 mm. In all patients, an intra-aortic balloon pump (IABP)was inserted immediately after admission to cardiac surgery service. All patients were operated at a mean of 3.3 ± 2.9 days, and 4 within 24 h of the rupture (range 1 to 9 days post-VSD). In 5 cases, the VSD was located superiorly, and 4 cases in the posterior septum.

**Results:**

The overall 30-day mortality was 11% (1/9). Coronary angiography was performed in all nine patients, four with single vessel disease had coronary stents implanted, and the other five received concomitant coronary artery bypass grafting during VSD repair surgery. There was no death in all 5 patients with anterior septal perforation. One patient with posterior septal perforation died in the operating room due to bleeding from the ventriculotomy site. Three survived patients were diagnosed with a small residual defect and mild left to right shunt post-repair. However, no further intervention was required, and patients remained asymptomatic (Killip II in 1 and III in 2).

**Conclusion:**

In our experience, immediate insertion of IABP and hemodynamic stabilization with early surgical intervention of VSD repair and concomitant coronary revascularization provided an 89% survival rate.

## Introduction

Mechanical complication with catastrophic cardiac rupture is the second leading cause of early mortality in patients with acute myocardial infarction (AMI) [1, 2]. Catastrophic cardiac rupture can be classified into three types; papillary muscle rupture, ventricular free wall rupture, and ventricular septal rupture. The incidence of papillary muscle rupture is rare and patients with ventricular free wall rupture generally die rapidly and rarely survive to undergo surgery. Patients with post-MI ventricular septal defect (VSD) can generally survive long enough to present to hospital, despite the severe hemodynamic disturbances with left to right shunt and cardiogenic shock that ensues [3]. Yet, their prognosis remains poor and their optimal management is uncertain [4].

Despite advances in medical and surgical therapies, as well as circulatory support, such as extracorporeal membrane oxygenation (ECMO) and ventricular assist devices (VAD), which allow the maturation of recent ischemic myocardium before surgical or percutaneous VSD closure, the mortality of post-MI VSD has remained very high [5]. For that reason, some recommended a delayed approach that will enable the natural selection of survivors who are likely to survive surgical repair. Therefore, improving the survival of post-MI VSD has important clinical implication to reduce the overall mortality of AMI with mechanical complications. Conservative treatment of patients with post-MI VSD, however, is associated with extremely poor clinical outcome with a 1-month mortality approaching 94% [6]. On the other hand, the early mortality for those opting for surgical treatment exceeds 40% and has not decreased in the last 20 years [7]. Surgical intervention still effectively improves the survival compared with conservative treatment, although the mortality remains far from acceptable. Due to limited resource in mechanical circulatory support technology in our institution, we adopted a moderate approach to correct the fundamental pathophysiology as soon as the patient is stabilized. Here, we review the outcomes of this surgical approach in patients presenting with post-MI VSD.

## Methods

### Data collection and ethics statement

A retrospective analysis of 9 consecutive patients who underwent emergency surgical repair of post-MI VSD was carried out at the surgical department of Shaanxi Provincial People’s Hospital in Xi’an, China. The patients’ demographics, peri-operative data, post-operative course and outcome were retrospectively obtained from chart review, and collected in the electronic database. The study was approved by the hospital Human Research Ethics Committee in July 2021 (No.2,021,049). The requirement for written informed consent was waived due to the retrospective nature of this analysis.

### Surgical techniques

A full median sternotomy approach was used in all cases. Cardiopulmonary bypass with moderate hypothermia and intermittent antegrade cold crystalloid cardioplegia were used for myocardial protection. Coronary artery bypass grafting, if needed, was first performed followed by VSD repair. All cases were performed with the infarct exclusion method using a bovine pericardial patch, described by David et al. [8]. Depending on the location of the VSD, a left ventricular incision was made through the infarcted area. The bovine pericardial patch was tailored generous enough, usually 1.5 cm wider than the VSD margin, to provide sufficient support to anchor the suture in healthy muscles without tearing. Continuous 4 − 0 polypropylene sutures were first used to sew the patch onto healthy myocardium 1.5 cm away from the perforated margin. The suture was kept in proper tension to avoid myocardial tearing caused by the fragile myocardium. Additional interrupted 4 − 0 pledgeted polypropylene sutures were used to reinforce the VSD repair. In patients with anterior VSD, the ventriculotomy incision was closed directly with a long-strip of felt and 2 − 0 polypropylene sutures. In patients with posterior VSD, we used a triangular-shaped bovine pericardial patch to close the incision that prevented tension on the fragile post-MI myocardium.

### Statistical analysis

Continuous variables are reported as mean ± standard deviation and compared using two sample t-tests. Categorical variables are summarized with percentages ± standard deviation. Statistical significance was defined as p < 0.05. Data were analyzed using SPSS version 22.0 (IBM, Armonk, NY,USA).

## Results

### Patient demographics and characteristics

From January 2013 to December 2020, 9 patients with a diagnosis of post-MI VSD were referred to the cardiac surgical department at Xi’an People Hospital. Of the 9 patients, 8 were male and 1 was female, ranging in age from 50 to 71 years-old (mean age was 58 ± 7 years). All patients underwent coronary angiography within 0 to 11 days of their ST-segment elevation myocardial infarction (STEMI). Five patients showed significant single vessel disease, 1 double vessel disease, and 3 patients had triple vessel disease. Coronary stenting was performed in 4 of these cases with single coronary artery disease,and the other five received concomitant coronary artery bypass grafting during VSD repair surgery. All patients were in congestive heart failure (Killip IV), requiring inotropic support. All patients received a small dose of norepinephrine 0.02–0.05 µg/kg-min intravenously. An intra-aortic balloon pump (IABP) was implanted into all patients upon transfer to the cardiac surgical unit. None of the patients needed intubation or mechanical ventilation. In all cases, the diagnosis was confirmed by transthoracic echocardiography (TTE). The surgical plan started with a reconstructed 3D heart model based on cardiac computed tomography (CT) data, which confirmed the position and structure of the septal perforation. The patients were operated on an emergency basis once the above preoperative preparation is completed, and the patient’s hemodynamic stabilized. The median interval between myocardial infarction and septal rupture was 4 days. The mean time interval from the diagnosis to operation was 3.3 days (1–9 days). Surgery was performed as soon as the consent was obtained. (Table 1)

**Table 1:** Patient Demographics and Characteristics

Cases	9
Age(years)	58 ± 7
Sex (female : male)	1:8
Acute PCI (n)	4
1/2/3 vessel disease	5/1/3
Anterior /Inferior VSD	5/4
VSD size	22.6 ± 5.7 mm 15–19 mm(n = 2)
	20–33 mm(n = 7)
Pre-op. IABP (n)	9
Pre-op. Mechanical ventilation (n)	0
VSD-operation interval	≤ 24 h (n = 4)
	2d-6d (n = 4)
	9d (n = 1)
Cardiopulmonary bypass time Aortic cross-clamp time	331 ± 81 min 193 ± 49 min

Values are presented as mean ± SD or the exact number. PCI = Percutaneous coronary intervention; VSD = Ventricular septal defect; Pre-op = Preoperative; IABP = Intra-aortic balloon pump

### Outcomes

Eight patients underwent emergency surgery within 1 week of post-MI VSD diagnosis. Four patients had surgery within 1 day, 4 between two to six days, and 1 on the ninth day after diagnosis due to delays in family decision. The median interval between diagnosis of VSD and surgery was 3 days (range 1–9 days). Five patients underwent concomitant bypass surgery at the same time, and the average number of distal anastomoses was1 ± 1.0 (range 0–2). The cardiopulmonary bypass (CPB) time for the posterior VSD group were significantly longer than those for the anterior group, (393 ± 85 min vs. 281 ± 28 min, p = 0.03). The mean aortic clamp time for the posterior VSD group tended to be longer than those for the anterior group but did not reach statistical significance(226 ± 18 min vs. 166 ± 51 min, p = 0.07).One patient with posterior VSD died intraoperatively due to uncontrolled bleeding from left ventriculotomy incision in our early experience. The remaining 8 patients stayed in ICU hemodynamically stable without any support. The heart function of these 8 patients at discharge was 2 in Killip III, 4 in Killip II and 2 in Killip I. These two patients with killip III were managed satisfactorily with diuretics. Three patients had small residual VSD with left to right shunts not requiring any further intervention. (Table 1) Eight patients who survived the operation in this cohort were discharged home without any major complications.

**Table 2:** Demographic, peri-operative data and outcome

	Sex	Age (years)	Time from AMI to VSD (Days)	Time from VSD to Surgery (days)	XC-time (min)	CPB time (min)	Operation			VSD			Residual Shunt		Killip	
							VSD repair	CABG	Location		Size (mm)	Size (mm)				
1	M	62	2	4	234	326	Y	2	Anterior		25			II		
2	M	53	2	1	152	259	Y				20			I		
3	M	50	4	9	206	285	Y				20			II		
4	M	59	11	1	133	281	Y	2			15			II		
5	M	53	8	1	109	257	Y				15			I		
6	M	71	7	2	249	493	Y	2	Posterior		25			expired		
7	F	52	0	5	207	326	Y				25	5		III		
8	M	52	3	6	216	319	Y	2			25	6		II		
9	M	66	2	1	233	435	Y	1			33	7		III		

M = male, F = female, XC-time = cross-clamp time, CPB = cardiopulmonary bypass, VSD = ventricular septal defect, CABG = coronary artery bypass graft

## Discussion

VSD remains a common mechanical complication after acute myocardial infarction, with an estimated incidence of 0.17% to 0.91 [5, 9]. More importantly, the pathological changes at the rupture site were different which manifested increased hemorrhage at the VSD site [10]. The survival outcome of conservative treatment is very poor with a 30-day mortality of over 90% [2, 6]. Since Cooley et al. reported their series of post-MI VSD repair in 1957, surgical management has made great progress. Nevertheless, the long-term outcome remains far from satisfactory. The reported mortality in the United States, if repaired within 1 week, is 54.1%, which constitutes one of the greatest mortality rates in the modern era of cardiac surgery [11]. For this reason, many surgeons prefer non-surgical intervention but rather stabilize the patient with circulatory support that allows natural selection of likely survivors. If they survive, surgical repair or percutaneous VSD closure can be performed. Therefore, the early surgical repair has drastically diminished over the last decade, from an initial 16.72–2.33% [5]. In the United States, only 7% of post-MI VSD patients received surgical intervention that is far less than the expected incidence of 0.3% in more one million AMI patients [5, 12]. Similarly, less than 12.0% of post-MI VSD were treated surgically in China [2].

The current ACC/AHA guideline recommends that all patients should undergo emergency operation unless there is a clear contraindication [13]. Whether early surgery will bring greater benefits to post-MI VSD patients is still controversial. Many have adopted a strategy of wait-and-see by temporizing hemodynamic instability while allowing the VSD to mature using various forms of circulatory support, and only then proceeding with surgical or percutaneous VSD closure if patients survive [14].

We believe that early surgical intervention will improve the overall survival of this challenging and fatal complication of AMI [10]. The interval from the onset of VSD to surgery is an important predictor for survival, although the optimal timing of surgery is still debatable [3, 7, 15]. The surgical mortality within one week after rupture ranged from 20 to 85% and markedly decreased to 0–16% after 4 weeks [16–19]. Nevertheless, those patients who died during the 4 weeks period were not considered in their surgical mortality. Several groups have proposed a delayed surgical strategy after initial medical therapy and IABP/circulatory support of the acute phase of MI, usually performing repair 4 weeks later. The reason behind this delayed approach is that fibrosis will normally matured after 3–4 weeks after an MI [14, 20]. The fibrotic boundary with the surrounding healthy myocardium will provide good tissue to withstand the strength of VSD repair. Therefore, it minimized the possibility of fatal bleeding and residual VSD caused by the hemorrhagic and necrotic tissues. This strategy does reduce the surgical mortality significantly, as long as the patients survives the natural selection [14, 19, 20]. This delayed strategy inherently has a selection bias, in that those patients with large VSDs or persistent hemodynamic instability or shock, will likely die before the optimal time to undergo the repair [21].

The high mortality in patients with post-MI VSD is not only due to the recent MI with ongoing ischemia, but the additional left-to-right shunt which further decreases cardiac output while overwhelming the normally low-pressure right heart with additional volume overload. In addition, the persisting high flow across the VSD with necrotic myocardium may increase the size of the rupture unpredictably. Even clinically stable patients may experience sudden hemodynamic deterioration and worsening cardiogenic shock. Once that happens, the surgical and natural mortality will increase significantly regardless of any interventions. Therefore, only less than 5% carefully selected hemodynamically stable patients survive delayed VSD repair [22]. This has led some to suggest that post-MI VSD should be operated as soon as diagnosed to improve overall survival [3, 10]. Lundblad et al. suggested to operate all post-MI VSDs upon diagnosis, and 85% of which were performed within one day. They reported a 30-day mortality rate of 33% [23]. Furukawa et al. reported no hospital mortality in all their 12 patient series with urgent surgical approach [24]. Among the 12 patients, 8 received an emergency operation, 2 within a few days, 1 in 25 days, and 1 in 58 days. Martinelli et al. on the other hand, operated on12 cases within a mean interval of 4 h without any surgical mortality [25].

The optimal timing for VSD repair is indeed a matter of debate in the field, and it seems that 7 days after perforation is a cut-off point [11]. Recent studies arguing that a delay strategy should be used, and that an emergency surgical strategy should only be used if patient was hemodynamically unstable [7, 19]. Another school of thought represented by Dr. David [26] et al. constantly suggested that an emergency surgery should be considered even if the patient is hemodynamically stable, unless there is a clear contraindication to surgery. Our current study seemed to be in agreement with the later approach.

Since 2013, our center took the strategy to operate on these patients once the diagnosis was confirmed, patient was hemodynamically stabilized, and consent obtained. The largest VSD diameter in our consecutive cohorts was 33 mm and the minimum diameter was 15 mm. With the exception of one patient who was operated on the 9th day, all the patients were operated within one week of diagnosis. All but 1 patients survived and discharged from the hospital with a mortality rate of 11.1%. The surgical mortality of post-MI VSDs varies greatly between different centers and surgeons, ranging from 20 to 100%, illustrating the challenges and difficulties of surgical intervention [26]. The main technical challenges for early surgery is the extremely fragile myocardium at the site of VSD caused by the severe myocardial edema and hemorrhage from AMI. The lack of obvious demarcation between healthy myocardium and necrotic myocardium, both lead to bleeding and residual shunt due to gradual tearing. The incidence of residual VSD has been reported to be more than 12% causing high perioperative mortality and heart failure [7].

Since 1987, the infarct exclusion method pioneered by David et al. has become the preferred surgical treatment of post-MI VSD with improved outcomes [8]. By isolating the infracted VSD area and free-wall myocardium provided healthy myocardium to anchor the patch, thus avoiding the excessive suture tension and tearing of the repair. This effectively reduces the recurrence of residual shunt, better preservation of the left ventricular geometry, and translates to better survival. It is important to emphasize that the suture line should be anchored in the healthy myocardium to prevent tearing and residual shunt. In our experience, the sutures should be placed at least 1.5 cm away from the VSD margin. Lundblad et al. compared the early and late results of using the infarct exclusion method versus the Daggett’s direct suture method. They claimed that the infarct exclusion method described by David et al. is safer and more conducive to avoiding post-operative residual shunt [27]. The higher ventricular pressure on the patch surface is always from left to right during systole or diastole. The patch is, thus pressed against the ventricular septum with no reverse force throughout the cardiac cycle. We preferred using a single running suture to fix the patch tightly. Then used additional interrupted pledged sutures to reinforce the suture lines. Some advocated the use of bio-glue between the patch and the edge of myocardium may reduce residual shunt [22].

Whether concomitant coronary artery bypass grafting (CABG) is indicated at the time of VSD repair is controversial [7, 28, 29]. Some studies suggested that the necrotic myocardium cannot be recovered by revascularization, and the concomitant bypass will only increase the ischemic time with minimal benefits [29]. Labroussea et al. reported that the concomitant CABG does not increase the risk of surgery and that hospital mortality is low [18]. As the incidence of multi-vessel disease is as high as 40% in post-MI VSD, coronary revascularization will not only help myocardial recovery in the short term but also reduce the risk of ischemia-related events and significantly improve long-term survival and quality of life [18] Barker et al. reported that the 4-year survival rate of VSD repair with CABG was 82.8% while the group without concomitant CABG was 32.2% [30]. As an institution, we are in favor of bypassing any coronary arteries with significant stenosis especially for patients with long life expectancy.

We also preferred to insert an IABP routinely in all patients because it can increase coronary perfusion and cardiac output while reducing cardiac afterload, so much so that it will reduce left to right shunt and volume overload to the right heart. We believe early initiation of IABP support, rather than waiting until hemodynamic instability, is more beneficial to VSD patients as it minimizes the hemodynamic effects of left to right shunt [31, 32]. However, some consider the use of mechanical circulatory support only when medical management fails. Yam et al. also advocates to implant IABP before surgery and believed that it would provide adequate hemodynamic stabilization and survival benefits [33].

Our data is a retrospective observational single institution experience of 9 consecutive patients who presented with mechanical complication of AMI. This study is limited by its small sample size and potential delay of surgical intervention due to few logistical reasons. The retrospective nature of the study will only allow us to analyze the result of our institutional approach with potential flaws and biases, and this study remains observational and non-randomized in nature.

## Conclusion

In our experience, immediate insertion of IABP and early surgical intervention of VSD repair with concomitant coronary revascularization within one week provided an 89% survival rate.

## Data Availability

The data used in the current study are available from the corresponding author.
