# Correction: Surgical repair of post myocardial infarction ventricular septal defect: a retrospective analysis of a single institution experience

**DOI:** 10.1186/s13019-024-02977-4

**Published:** 2024-08-10

**Authors:** Jian Shi, Jeremy Y. Levett, Haiyu LV, Guoan Zhang, Sha Wang, Tao Wei, Zhikun Wang, Xi Zhang, Dawei Feng, Kan Wang, Qiang Liu, Dominique Shum-Tim

**Affiliations:** 1 Department of Cardiac Surgery, Shaanxi Provincial People’s Hospital, Xi’an, China; 2 Division of Cardiac Surgery, Department of Surgery, McGill University Health Centre, McGill University, Montreal, QC Canada

**Correction: Journal of Cardiothoracic Surgery (2023) 18:313**

10.1186/s13019-023-02418-8.

Following publication of the original article [1], the author’s name ‘Jeremy Y. Levett’ was incorrectly written as ‘Jeremy Levett’.

The original article has been corrected.

## References

[1] Shi J, Levett J, LV H, et al. Surgical repair of post myocardial infarction ventricular septal defect: a retrospective analysis of a single institution experience. J Cardiothorac Surg. 2023;18:313. 10.1186/s13019-023-02418-8. 37950265 10.1186/s13019-023-02418-8 PMC10638688

